# Sex differences in neonatal brain injury and inflammation

**DOI:** 10.3389/fimmu.2023.1243364

**Published:** 2023-10-25

**Authors:** Lynne A. Kelly, Aoife Branagan, Gergana Semova, Eleanor J. Molloy

**Affiliations:** ^1^ Discipline of Paediatrics, Trinity College Dublin, Dublin, Ireland; ^2^ Paediatrics, Trinity Translational Medicine Institute (TTMI), Dublin, Ireland; ^3^ Department of Medicine, Trinity Centre for Health Sciences, Trinity Research in Childhood Centre (TRiCC), Dublin, Ireland; ^4^ Coombe Women and Infants University Hospital Dublin, Dublin, Ireland; ^5^ Neonatology, Children’s Health Ireland (CHI) at Crumlin, Dublin, Ireland; ^6^ Neonatology and Neurodisability, Children’s Health Ireland (CHI) at Tallaght, Dublin, Ireland

**Keywords:** inflammation, neonate, brain injury, neonatal encephalopathy, sex

## Abstract

Neonatal brain injury and associated inflammation is more common in males. There is a well-recognised difference in incidence and outcome of neonatal encephalopathy according to sex with a pronounced male disadvantage. Neurodevelopmental differences manifest from an early age in infancy with females having a lower incidence of developmental delay and learning difficulties in comparison with males and male sex has consistently been identified as a risk factor for cerebral palsy in epidemiological studies. Important neurobiological differences exist between the sexes with respect to neuronal injury which are especially pronounced in preterm neonates. There are many potential reasons for these sex differences including genetic, immunological and hormonal differences but there are limited studies of neonatal immune response. Animal models with induced neonatal hypoxia have shown various sex differences including an upregulated immune response and increased microglial activation in males. Male sex is recognized to be a risk factor for neonatal hypoxic ischemic encephalopathy (HIE) during the perinatal period and this review discusses in detail the sex differences in brain injury in preterm and term neonates and some of the potential new therapies with possible sex affects.

## Introduction

Neonatal brain injury is associated with long-term neurodevelopmental disability and is more common in boys. There are many factors, pre-, peri- and post-natal, genetic and metabolic factors, which can interact and result in brain injury in the neonatal period (1). Neonatal encephalopathy (NE) occurs in an estimated 3 in 1000 infants born at term and is a result of a wide variety of eitiologies (2) and the incidence is higher in preterm infants at 4–48 per 1000 preterm births (3) and preterm birth is the leading cause of neonatal mortality worldwide. The global incidence of NE is 1.15million (4) and approximately half of infants with NE will have adverse outcomes including cerebral palsy, intellectual disability, and learning disabilities. Sex differences are especially obvious in preterm babies with differences in crown rump length and placental gene expression (5), nutrition, growth and metabolism in males versus female infants (5).

Inflammation in the perinatal period results in the activation of the innate immune system and increased production of cytokines and some of the causes include chorioamnionitis, hypoxia-ischaemia and preterm birth (3). The activation of the immune system involves both systemic and cerebral inflammation that can result in brain injury and lead to persistent adverse effects on the developing brain (6).

Cytokines modulate inflammation and repair after inflammation-related brain damage. Altered cytokines have previously been described in infants with NE versus healthy control infants (4). The cytokines Interleukin-1 (IL-1), Interleukin-6 (IL-6) and Tumour necrosis factor-alpha (TNF-α) may be influential in the initiation of inflammation, recruitment of other cytokines and leukocytes. TNF-α, IL-1, IL-6, and IL-8 both attract and stimulate leucocyte adhesion, which cause immune reaction and activate phagocytosis (7). Increased circulating pro-inflammatory cytokines can, by crossing the blood brain barrier, activate microglia and astrocytes (8).

This ongoing inflammatory “hit” augments the activation of resident immune cells in a process which prolongs the original inflammation and activates a secondary process of brain injury. The innate immune cells of the brain, microglia, become activated, monocytes, macrophages and neutrophils become activated and increase the release of proinflammatory cytokines. The blood-brain barrier becomes compromised and cross-talk occurs with the peripheral immune system leading to an ongoing inflammatory response (9).

The tertiary phase of brain injury follows, spanning weeks to years after the initial insult and is characterized by persisting neuroinflammation, excitotoxity, and endogenous neuroregeneration and repair (10). Persistent inflammation has been described in children with cerebral palsy school-age children following preterm neonatal brain injury (11–13) which shows altered persistent systemic inflammation and may provide a window of opportunity in relation to anti-inflammatory therapies. MRI is the standard neuroimaging technique used to measure brain injury and a study by Ní Bhroin et al. showed MRI scoring systems in the first two weeks of life correlate with outcome at 2yrs of age in infants with NE (14).

## Sex and brain injury

Male infants are at a higher risk of adverse outcome compared to females and have higher rates of morbidity and mortality compared to their female counterparts. This marked sexual dimorphism for outcome and mortality is especially pronounced in preterm infants (15). There is a sexual dimorphism that exists with respect to neuronal injury and very premature males appear to be more vulnerable to white matter injury and intraventricular haemorrhage (IVH) than females. Important neurobiological differences exist between the sexes with respect to neuronal injury (16, 17). Male children have a higher incidence of neurodevelopmental delay, autism, attention-deficit-hyperactivity disorder, and cerebral palsy (18–20). However, the mechanisms underlying these sex differences are elusive and studies are still required to uncover the pathways and molecules involved. Some major pathways known to be involved through studies to date include hormones, oxidative stress, cell death and the activation state of microglia (21). There are many potential mechanisms for these sex differences although three key potential mechanisms of sex differences in inflammation and brain injury lie in steroid hormones, the X chromosome and inflammatory pathways. Sex hormones affect the immune response and may contribute to sex-differences in immune responses postnatally (22). 17-β Estradiol (E2) and progesterone (Pg) have been shown to have neuroprotective effects in rat models of brain injury with similar effects on male and female rat pups using E2 and more pronounced long-term tissue protection in males with Pg (23, 24). Despite a small number of animal studies, there are very few examining steroid hormone levels that stratify by sex leaving large gaps in our knowledge. Many of the immune related genes can be found on the X chromosome and naturally the XX phenotype of females confers a greater immune advantage to this sex, although is also the predominant cause of a higher incidence of autoimmune disease in females. The X chromosome is polymorphic and also in the female displays unique mosaicism which may play an important role in their innate immune response (25). There are also many studies on inflammatory pathways but little on systemic postnatal samples and although we know that male sex is associated with increased risk of neonatal infections further research data is required in this area.

## Preterm inflammation

There is a marked difference in immune response between males and females throughout life (26) with an increased male susceptibility to sepsis (27). Multiple clinical and epidemiological reports continue to suggest the strong association of both maternal immune system dysregulation and foetal inflammation with preterm birth, brain injury and adverse neurodevelopmental outcome (28–31). Neonatal sepsis is the leading cause of infant mortality with additional risks of adverse neurodevelopmental outcome with early- and late-onset sepsis (29, 32) and bronchopulmonary dysplasia (BPD) increasing in those with infection (33) and the risk of necrotizing enterocolitis (NEC) (34, 35). Vulnerability and susceptibility of the foetal brain to such inflammatory events can profoundly alter its natural development and selectively damage the white matter (36) which is the most commonly observed type of brain injury on routinely collected neuroimaging studies (37). Preterm infants also have reduced growth and complexity of cortical and subcortical grey matter structures at term equivalence compared with their term counterparts (38). Abnormalities in white matter have also been reported in preterm infants and microstructural changes on MRI could be related to neurological disability in later life (39). Many clinical and laboratory studies on neonatal immune function did not stratify by sex resulting in a paucity of immunological study on postnatal sex-differences in neonates.

Neonates largely depend on a developing immune system for mediating immune responses as their adaptive immune response and memory to pathogens develops rapidly over the first 3 months of life (40–42). Neonatal immune responses are generally skewed towards the Th-2 helper T cell responses, enabling immune tolerance to maternal antigens instead of providing defence from microbial infections (43, 44). A significant reduction in phagocytic activity and enhanced production of reactive oxygen species, defective neutrophil amplification and attenuated pro-inflammatory cytokine responses of monocytes after bacterial stimulation are all distinctive features of innate responses especially pronounced in preterm infants (38, 43, 45). In animal models, increased permeability of the blood-brain barrier (BBB) to pro-inflammatory cytokines, chemokines such as IL-1β and TNF-α and the direct leak of bacterial products such as lipopolysaccharide (LPS) and bacterial lipopeptide (BLP), that activate microglia to release inflammatory mediators, induce preterm brain damage. Results from animal models (38, 46, 47) suggest that prolonged postnatal inflammation as well as hyperoxia strongly attenuate oligodendrocyte cellular dynamics involving apoptosis and developmental arrest. Both, oligodendrocyte death and developmental arrest result in hypomyelination and a disturbed white matter microstructure.

Leviton et al. demonstrated that repeatedly elevated concentrations of inflammation-related proteins such as tumour necrosis factor-a (TNF-α), tumour necrosis factor-a-receptor-1 (TNFR1), interleukin-8 (IL-8), intercellular adhesion molecule-1 (ICAM-1), interleukin-6 (IL-6), E-selectin, and insulin-like growth factor binding protein-1 (IGFBP-1), during the first 2 postnatal weeks (48) led to an increased risk of a cerebral palsy diagnosis 2 years later (49). This group also described elevated levels of IL-6 and IL-8 and, to a lesser extent, TNF-α and IL-1β measured during the first 3 weeks of life that most consistently predict poorer performance across neurodevelopmental outcomes (50, 51). Additionally, these authors reveal that persistent inflammation, three weeks post birth, more reliably predicts long-term neurodevelopmental impairment than transient inflammation and suggest that assessment of systemic inflammation and neurodevelopment should include multiple measures rather than relying solely on single time point measure of elevated blood cytokines/chemokines. Further, work by Zareen et al. on persistent inflammation in children post-NE at birth has shown increased IL-2, IL-6, IL-8, TNF-β and GM-CSF levels correlates with neurodevelopmental outcomes (12).

## Systemic inflammation in the term infant

Neonatal encephalopathy (NE) is the most common cause of neonatal brain injury occurring in term and near-term infants >35 weeks gestational age (52). NE encompasses many possible aetiologies including hypoxic-ischemic (HI) injury, perinatal infections, placental abnormalities, metabolic disorders, brain malformations, vascular injuries (including stroke) and other causes (2, 53). The pathophysiology of NE can be divided into three distinct phases (54). The primary energy failure phase occurs after initial insult due to decreased cerebral blood supply (55). The latent phase occurs after cerebral reperfusion. Reperfusion will trigger a secondary injury between 6 and 72 hours related to secondary energy failure including depletion of adenosine triphosphate (ATP) reserves and production of lactate and reactive oxygen species (ROS) and mitochondrial dysfunction. The majority of cell death and clinical seizures occur during this period (56). The tertiary phase involves persistent inflammation and epigenetic changes that persist over months and years (10, 12, 57).

Inflammatory molecules secreted to allow communication between cells have been shown in multiple studies to be altered in infants with NE (58, 59). In the context of neonatal brain injury, these will activate cytotoxic T cells and natural killer cells, the clinical endpoint of which is white matter changes and neurodevelopmental impairment. IL-6, IL-8, and vascular endothelial growth factor (VEGF) have been seen to be higher in infants with NE at varying timepoints after brain injury, when compared to term controls (59, 60) and also in infants with severe NE compared to the milder phenotype (57, 61, 62). O’Dea et al. have published studies on cytokines and their correlation with MRI and developmental outcomes in term infants with neonatal encephalopathy. This study examined, at baseline and also upon endotoxin stimulation, pro and anti-inflammatory cytokines and showed that infants with NE have an altered inflammatory state compared to control term infants which may prove important in further management of these infants, as understanding these cytokine responses will underpin the development of new adjunctive therapies in the future (4, 62, 63).

## Sex in preterm infants and brain injury

Clinical studies in preterm and term infants are less frequently reported to animal studies on sex differences in brain injury, a recent review article details sex specific differences in brain injury and repair in infants (64). Inflammatory changes are also evident in preterm infants, pro-inflammatory cytokines such as Interleukin-1B (IL-1β) and Tumour necrosis factor alpha (TNF-α), among others, have been shown to have increased expression after neonatal brain injury (65). Smith et al. found higher behavioural scores in premature females and in postnatal day 7 rodents, male deficits in behavioural tasks including spatial and non-spatial memory tasks, rapid auditory processing tasks and performance IQ were significantly different to that of the female (66). Research has also reported significant differences in the incidence and severity of respiratory distress syndrome in infants and in a study of very low birth weight infants the main outcomes measured, mortality or major morbidity including bronchopulmonary dysplasia (BPD) and necrotizing enterocolitis (NEC), were higher in males than females (67). Animal studies with the Rice -Venucci model (68) has uncovered sex differences in behavioural tasks of a preterm rat model with male deficits (66) while female microglia have a more robust immune response to neuronal injury and a higher level of neurogenesis in term rodents. The male mice in this study also displayed more cognitive defects compared to females following similar brain injury in both sexes (69). Sanches et al. showed marked structural differences in the brain structures between the sexes in postnatal rodents (70). O’Driscoll et al. described sex differences in outcome in preterm infants with males having poorer outcomes and a higher susceptibility to pulmonary hypertension, respiratory distress syndrome (RDS) and inflammation (15, 22). Preterm males have a higher incidence of Intraventricular haemorrhage (IVH) and periventricular leukomalacia (PVL), and increased severity of brain lesions with follow-up data suggesting that major cranial abnormalities on neuroimaging are more common in males (18, 71). Sanches et al. demonstrated that histological damage was sexually dimorphic in specific brain structures (70) and after HI at postnatal day 3, (which mimics HI injury in preterm infants) female rats exhibited larger histological damage when assessed at adulthood (72).

Despite the higher incidence of such abnormalities in males, it has been suggested that extremely preterm male infants also have a higher risk of adverse neurological outcome in the absence of severe IVH or PVL (73). A recent meta-analysis on the male disadvantage has shown that preterm infants have an increased risk of severe IVH, PVL, BPD, NEC and retinopathy of prematurity (ROP) (74) and these complications can increase the risk of brain injury. Preterm males are reported to have greater proinflammatory responses, oxidative stress and increased infection rates and specific immune cell phenotypical differences (22).

## Sex in term infants and brain injury

**Figure 1:**
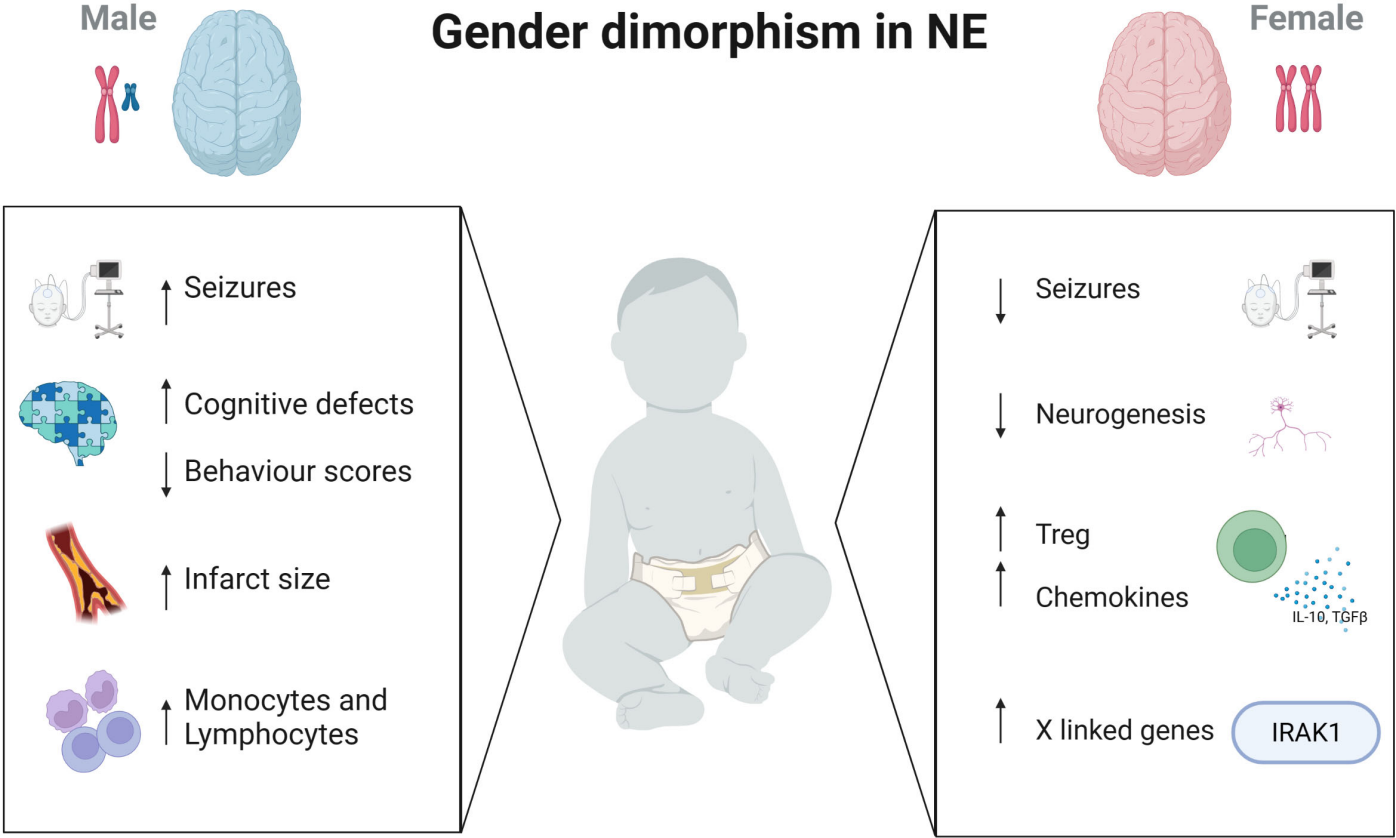
Immune differences in males and females with brain injury. Sex differences presents in many aspects of the brain. While male neonates have increased seizure burden, cognitive defects and larger infarct sizes, females have increased Treg cells, chemokine levels and immune-regulating genes on the X chromosome. Created with BioRender.com.

Neonatal encephalopathy is a complex disease occurring in the perinatal period caused by a number of aetiologies such as hypoxia ischaemia, perinatal infection, neonatal stroke, infection and placental abnormalities (2, 75). In terms of males and females, much of our knowledge of sex differences lies in animal models. Many animal studies have used the Rice-Vanucci model of HIE to induce neuronal injury and have reported results mainly in preterm but also in term rodents. In mice sex differences in brain injury with early activation of caspase-dependent pathways in female mice were demonstrated and activation of inflammatory cascades in both sexes which then persists in female mice (76). In a term mouse model of HIE injury, female mice displayed less seizures and a smaller infarct size compared to males by day 3 post injury. Similarly, at this timepoint they showed sex differences in the levels of microglia and inflammatory markers (IL1-β and TNF-α) present between the sexes. There were significantly more monocyte and lymphocyte infiltration in male versus female brains 3 days after HIE (77). In a more recent study, regulatory Tregs from neonatal female mice provide endogenous neuroprotection with increased cerebral Treg infiltration, coinciding with elevated chemokine receptor expression. Depletion of regulatory T cells in these HI-induced mice aggravated brain tissue injury, short-term motor deficits, and long-term deficits in exploratory activity in the female mice and endothelial activation and leukocyte infiltration (78) (**Figure 1**).

## Sex, inflammation and brain

Sexual disparities are prominent with respect to immune function and preterm birth (15, 22). There is a more robust sepsis response in female neonates, that male neonates are more commonly affected, they have distinct endotoxin responses and hormones may modulate their immune responses (15). Male sex is associated with increased risk of almost all forms of commonly encountered neonatal infections including community-acquired sepsis (79), group B streptococcus (GBS) sepsis (80) and sepsis secondary to drug-resistant or atypical bacteria (81). In addition, sepsis related mortality in males is higher than females (82–84). There is a more pronounced proinflammatory state among males in human studies where umbilical cord blood from male infants exhibits higher IL-6 and IL-1β following endotoxin stimulation than females (85).Females may have an immune advantage conferred by their XX genotype (86). Several genes encoding key metabolic and regulatory proteins reside on the X chromosome, including members of the apoptotic cascade, hormone homeostasis, glucose metabolic enzymes, superoxide-producing machinery, and the toll-like receptor/nuclear factor B/c-Jun N-terminal kinase signalling pathway. The X chromosome encodes many immune related genes and this may confer females with an immune advantage over males. Some genes on the X-chromosome encode for several components of the Toll like receptor (TLR)-4 pathway that are essential for Nuclear factor kappa B (NFκB) signaling e.g Interleukin 1 receptor associated kinase 1 (IRAK1) and a subset of genes with specific roles in innate immunity. The main function of IRAK1 is its critical role in regulating an immune response against pathogens. IRAK1 is expressed at higher levels in females versus male neonates (87). Another possible mode of immune activation in the brain is the role of TLR7, a key regulator of innate and adaptive immune responses, which is highly expressed in females in contrast to males, males also display higher angiotensin-converting enzyme 2 (ACE2) than females and the nuclear fertility restorer gene (RF5) which drives interferons, is higher in females. Klein et al. (26) detail the distinct differences between males and females and their immune responses with differing numbers of innate immune cells, receptors and cytokines and distinct differences in adaptive immunity between males and females and also detailed by Lenz et al. (88) specifically the brain and microglial differences and development and by Mallard et al. (89), as such the detail of these sex differences in the immunes cells will not be expanded further within this review.

## Potential treatments affected by sex

Modulating innate immune responses may prove to be vital in the search for adjuvant treatments to improve outcome – medications under investigation at present include allopurinol, melatonin and erythropoietin (1). The ongoing inflammatory response following HI results in a secondary stage of inflammation which may be vital in immunomodulation therapy. There is mounting evidence that suggests regulating the immune response will provide more positive outcomes in cases of brain injury at birth. A recent study in rat pups have shown that of 25 potential new neuroprotective agents, eight of these significantly improved brain area loss following HI injury (90). We have detailed some current therapies in neuroprotection trials and review those showing sex specific responses for preterm and term infants.

## Preterm infant therapies

### Caffeine

Clinical studies have shown that caffeine has neuroprotective effects in premature infants by alleviating hypoxia-induced white matter damage, and by improving ventilation function and brain self-regulation (91, 92). In addition, caffeine has been shown to reduce the apoptosis of developing brain neurons, ventricular enlargement, and white matter loss caused by hypoxia (93). Animal models of preterm birth show the beneficial effects of caffeine on brain injury (94) and one study reported that intermittent hypoxia significantly increases the number of apneas in male but not in female preterm rat pups. Moreover, caffeine and erythropoietin (EPO) in males prevented the increase of apneas induced by intermittent hypoxia, and the administration of both drugs together did not provide a cumulative beneficial effect. These effects were not seen in the comparative female group (95). Concerns for caffeine use have arisen with one high dose trial reporting statistically significant increases in abnormal neurological outcomes compared with standard dose (96). Results from a randomised double-blind placebo controlled study showed the initiation of early caffeine did not reduce the age of first successful extubation, rates of BPD, or the duration of need for supplemental oxygen when compared with the placebo group and this trial was halted due to a non-significant trend towards an increased mortality rate in the caffeine group (97).

### Indomethacin

The Trial of Indomethacin Prophylaxis in Preterm Infants (TIPP) used low-dose indomethacin on very low birth weight infants to determine if improvement in survival without cerebral palsy or developmental problems at 18 to 22 months of age could be achieved. The study results show that indomethacin reduced the incidence of patent ductus and of severe periventricular and intraventricular haemorrhage (98). Ment et al. suggested that indomethacin reduced white matter damage through an anti-inflammatory effect that is more pronounced in the more vulnerable male cells and halved the incidence of intraventricular haemorrhage, eliminated parenchymal haemorrhage, and was associated with higher verbal scores at 3 to 8 years in boys (99). A secondary analysis was performed in the entire TIPP cohort suggested a weak differential treatment effect of indomethacin by sex. Using a composite of outcome variables including, death and one or more of cerebral palsy, cognitive delay, blindness and deafness, the authors found that the effect of indomethacin differed between males and females (p=0.048). This primary composite outcome of death or severe neurodisability was more frequent in males than females, with more male deaths or survival with impairment, in particular those treated with indomethacin (100). More recently though this study has shown that the added prophylactic indomethacin resulted in a higher frequency of the primary outcomes of death or severe neurodisability than placebo (101).

### Estrogen and analogues

Trotter et al, investigated the use of 17β-Estradiol and Progesterone in female preterm infants. These hormones rise dramatically in the final trimester and preterm infants lack this exposure. The initial scope of the work was to investigate if these hormone levels could be replenished with exogenous hormones (102). A randomized trial was conducted on the benefits of estradiol supplementation in preterm infants (n=30), only female infants were included as uterine growth was selected as the indicator for the biological effectiveness of the E2 and Pg replacement. This study found E2 and Pg replacement *via* IV and trans-epidermal routes can maintain plasma levels as high as those *in utero* with biological effectiveness (103). A further study on replacement E2 and Pg in preterm infants included both male and female infants and found no benefit in replacement therapies for the incidence of BPD and death (104). Follow up on these infants, with development outcome measured, reported a higher psychomotor score, within normal range, on the Bayley Scales of Infant and Toddler Development (BAYLEY II) scale in the treated group compared with the below average score in the untreated control group which shows the potential for sex steroids to benefit the preterm developing brain (105–107).

## Term infant therapies

### Therapeutic hypothermia

Therapeutic hypothermia (TH) is the only available treatment in cases of neonatal encephalopathy. However, studies show it is effective in only 50% cases and adverse outcomes such as death, neurodevelopmental delay and severe disability occur in in the other half of cases (108). Recent data suggests that therapies may need to be sex-specific to have their maximal effect and the best possible outcome in these infants. TH has not been evaluated in a sex-specific manner in the treatment of perinatal asphyxia (109, 110), neither have more recent studies into the evaluation of this therapy (111, 112). More recently, the use of TH and sex differences has been reviewed by combining several animal studies from one centre and showed that the effect of TH on neuroprotection was greater in females than males (113).

### Allopurinol

Allopurinol is commonly used in the treatment of gout in adults. Animal studies have shown some promising data in relation to its potential in reducing the level of brain injury following perinatal asphyxia by reducing the number of oxygen radicals and combined with TH may benefit the neurodevelopmental outcome (114). In a Cochrane Systematic review, Chaudhri (2012) found the three RCTs on this drug to have insufficient data with no clinically significant beneficial effects of combined TH and allopurinol as the patient numbers were too small n=114 (115). The ongoing randomised control trial, Effect of Allopurinol for Hypoxic-ischemic Brain Injury on Neurocognitive Outcome (ALBINO) trial is underway by Maiwald et al. which will analyse the safety and efficacy of allopurinol combined with TH to improve outcomes (114). There are few studies which take sex differences into account although research has shown that male infants have worse outcome than females. In a rodent model of TH with allopurinol dual therapy confers greater neuroprotection than TH alone after a HI injury. The improvements found, both at the molecular level and histologically, were more important in females than in males (116).

### Zenon gas

Zenon is a noble gas with an excellent safety profile in animal studies (117). Its use was investigated in combination with TH and in a rat model showed that there was functional improvements that were greater than in the TH alone group and that this was sustained over time (118). This is one of the only studies which could be found in relation to sexand adjunctive therapies to TH as on further exploration of this study, the authors found that females have better motor scores following this treatment than males but no change on histology (119). No studies have yet shown that Zenon is an affective adjuvant therapy in infants with HI at birth.

### Further potential - therapies in trials

There are various other therapies being designed to improve outcome for neonates with brain injury. In combination with TH, these therapies may play an important role in altering the inflammation seen in these infants as inflammation plays a critical role in the pathogenesis of neonatal brain injury. Erythropoietin (Epo) has anti-inflammatory, anti-oxidant and anti-apoptotic effects and promotes neurogenesis and angiogenesis. There are trials underway to determine its potential as a therapeutic agent in preventing adverse outcome in neonates with brain injury and inflammation. The Preterm Erythropoietin Neuroprotection Trial (PENUT) is a Phase III of high dose Epo for neuroprotection in the preterm infant that looked at a sex effect and found no difference in the treatment groups in relation to sex (120). A phase III trial, the High-Dose Erythropoietin for Asphyxia and Encephalopathy (HEAL) trial, recruiting term or near term infants with moderate or severe HIE has recently published their data and reported that there was no added benefit to the administration of Epo with TH than TH alone. This study found that the additional treatment of EPO did not result in lowering the risk of death or neurodevelopmental outcome than placebo and was also associated with a higher rate of serious adverse events (121). Further therapies are in trials for the treatment of neonatal brain injury such as cell-based therapies e.g mesenchymal stem and autologous umbilical cord cells (122, 123) and N-acetylcysteine (124), melatonin (125) and pentoxifylene (126). To date we could not source information on these treatment effects and sex differences, but it is important to consider this as a variable in the treatment of neonates with brain injury. Males and females differ in respect to immunotherapies in the setting of cancer, autoimmune disorders and infectious disease (127) and these differences may also translate into paediatric disorders therefore individualised care must be considered with current therapies on trial.

## Conclusions

There are gaps in our knowledge within this topic. The first of these is that many clinical and laboratory studies have not reported results by sex, therefore limiting our insights into the potentially important differences in neonatal immunity between the sexes, future studies would benefit in reporting results with sex as a biological variable in all neonatal studies. Another important deficit in scientific investigation is the lack of study on postnatally acquired samples in neonates. At present much of our human data is from umbilical cord blood which, while easily obtainable, does not mirror the more clinically important postnatal immune system. There are a multitude of studies that show clear differences between males and females in their brain structure, response to hormones and response to treatments in terms of brain injury. The mechanisms responsible for these observed differences are still elusive. In terms of care of the newborn, particularly those with HI, it must be of importance in the treatment and interventions used, to take this sexual dimorphism into account to have optimal outcome for the infant (128, 129). Research funding bodies are now aligning to this and there are new requirements for research studies to consider sex as a variable in their design and interpretation. Clinical trials are underway on new therapies that will potentially complement TH, reduce the morbidity and mortality rate and improve neurodevelopmental outcomes in these infants while hopefully taking into account the reported immunological sex differences observed.

## Author contributions

LK and EM conceptualization. LK, AB, GS, EM writing original draft, LK and EM review and editing. All authors contributed to the article and approved the submitted version.
